# Supplementary figures and images for: Does education sculpt healthcare choices? Exploring factors influencing healthcare utilization among female youths in eight low and lower-middle-income countries
Source: PLoS One. 2025 Mar 17;20(3):e0298782. doi: 10.1371/journal.pone.0298782 (PMC11913268; doi:10.1371/journal.pone.0298782)

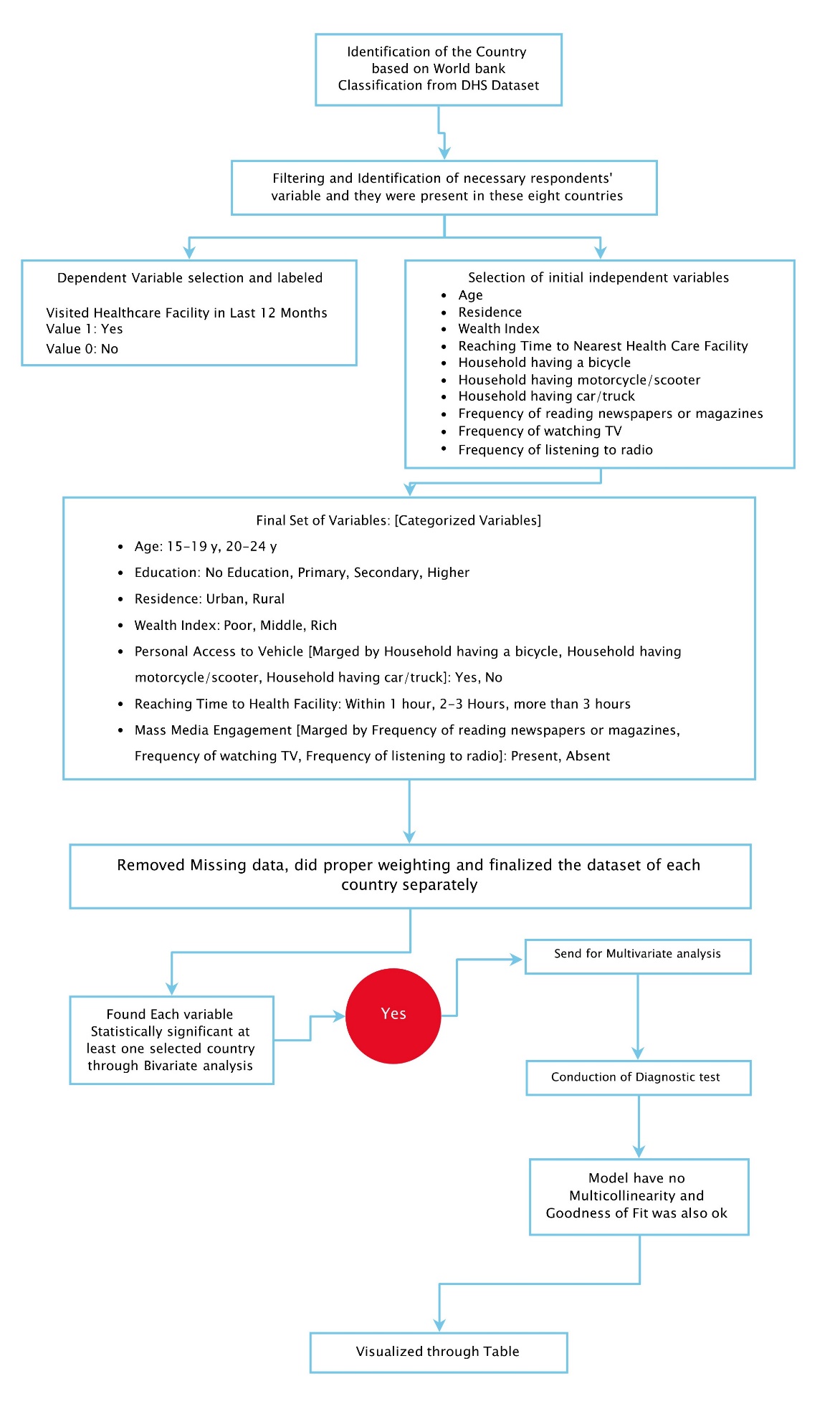


Supplementary Figure 1. Flowchart of data selection and analysis process

Supplement: S1 Fig — Flowchart of data selection and analysis process. (DOCX) [file pone.0298782.s001.docx]
