# Supplementary material for: Does education sculpt healthcare choices? Exploring factors influencing healthcare utilization among female youths in eight low and lower-middle-income countries
Source: PLoS One. 2025 Mar 17;20(3):e0298782. doi: 10.1371/journal.pone.0298782 (PMC11913268; doi:10.1371/journal.pone.0298782)
Supplement: Attachment — Submitted filename: Recommendation.docx [file pone.0298782.s002.docx]

- It is well known that education plays a significant role in healthcare usage across countries, and education positively influences it. Many studies mention that like (Rizvi DS. Health education and global health: Practices, applications, and future research. J Educ Health Promot. 2022 Aug 25;11:262. doi: 10.4103/jehp.jehp_218_22. PMID: 36325224; PMCID: PMC9621358). What did this study add to what was already known on this topic?
- The manuscript needs English editing
- The title is informative but so long. It is better to be considered
- Line 51: it is better to write ‘’Education significantly influences healthcare utilization, positively impacting both primary and secondary education’’
- The aim is clear
- What is the role of the researchers? Is that collected data from the Demographic and Health Survey (DHS). You did not collect data by yourself
- Are you sure the variables are defined and measured appropriately?
- Why did you not use a flowchart to explain the variables
- The results need to be discussed from multiple angles and placed into context without being overinterpreted
