# Supplementary material for: Does education sculpt healthcare choices? Exploring factors influencing healthcare utilization among female youths in eight low and lower-middle-income countries
Source: PLoS One. 2025 Mar 17;20(3):e0298782. doi: 10.1371/journal.pone.0298782 (PMC11913268; doi:10.1371/journal.pone.0298782)
Supplement: Attachment — Submitted filename: Response to Reviewers.docx [file pone.0298782.s004.docx]

Dear Reviewers,

Thank you for your insightful and constructive feedback on our manuscript. We have carefully considered your comments and made the following revisions:

**Reviewer 1:**

**Comment 1**: The article touches on an interesting and useful point in the context of the SDGs, tracking their progress and inequalities in access across SSA. I was surprised to see the choice of the outcome variable as a proxy for access to health overall. I would suggest the authors to research a bit more on what variables are usually used, from the DHS surveys, to analyze access to health facilities. The one chosen could lead to potential biases as it only reports accesses in the past month. Please explore other options and/or cite previous published studies where the same variable has been used for similar purposes.

**Response** **1**: Thank you for your insightful comment. However, we used the "Visited health facility in the last 12 months" variable, not just visits in the past month, as our outcome variable. This choice captures a broader time frame, reducing potential bias associated with short-term health-seeking behavior fluctuations. Similar variables have been successfully used in previous studies. For example, a study using the Medical Expenditure Panel Survey found that 56.3% of children aged 0 to 18 years had no well-child visits during a 12-month period, highlighting the importance of this time frame in capturing health-seeking behavior. Additionally, research focusing on long-term care services emphasized the significance of considering longer periods when assessing healthcare utilization, particularly among the aging population. Furthermore, studies examining trends in healthcare utilization beyond immediate periods, such as during lockdowns, reinforce the need for extended time frames to understand comprehensive healthcare-seeking behaviors. Therefore, we may say these examples support the appropriateness of using a 12-month period to evaluate healthcare utilization. We have also, added such a section in lines (137, and 146) and cited the necessary paper inside the manuscript.

**Comment 2**: No covariate selection process was done and an arbitrary choice of covariates for the model. Usually, from an initial literature review where a list of covariates is pre-selected based on literature, statistical techniques are used to select covariates that go into the final model.
-The choice of covariates should be driven by the statistical selection and not from the authors.

**Response 2**: We appreciate your concern. The selection of covariates was not arbitrary. Through literature review, we identified potential covariates and then we included covariates that showed a significant result in any of the countries we studied, with a minimum significance value of less than 0.05. If any variable was significant in any country we have chosen, it was retained and used in the final model. We have now additionally included this in the manuscript at (line 170). Thanks.

**Comment 3**: Testing for multicollinearity is mentioned in the text, however, no details were given (e.g. what tests were used / what cut-off was considered). Testing for interactions between covariates has not been performed or reported.

**Response 3**: Thank you for pointing this out. To address multicollinearity, we conducted a thorough diagnostic process. Given that we used the “SVY” command, we created a dummy variable to calculate the residuals by subtracting the predicted values from the observed values. We then generated a correlation matrix to examine relationships among the independent variables and the residuals, using a cut point of 0.5 to determine significant multicollinearity. The results indicated that the residuals were not correlated with any of the independent variables, confirming that multicollinearity was not a significant issue. Additionally, we performed the Hosmer-Lemeshow goodness-of-fit test with the group value set to 10, and the p-value exceeded 0.5, indicating a good fit. We have also added this to the manuscript (line 194) to avoid getting further comments.

**Comment 4**: Not clear from the text if the authors have pooled the data for all the countries together or not. In the case of a pooled dataset, I believe the recommendation from the DHS Program is to transform the weights (from normalized to de-normalized) before undertaking any analysis. This is because the sampling weights are country-specific, and therefore not comparable. I would strongly recommend the authors consult the DHS material on this matter and re-run the data if using a pooled dataset.

**Response 4**: We appreciate your concern for clarification. We did not combine data from all countries at once. Instead, we conducted separate analyses for each country and displayed the results in a table. The same variables and code were used for each country's analysis to ensure consistency. We have added this information, in the manuscript section at (line 180).

**Comment 5**: If I understand well the authors have used the svyset command in STATA to weight the data and take into account, the sampling structure, and included the weighting (see above for more about sampling weights). The authors have cited the DHS blog (ref 33) however the link does not work (at least on my hand). If working with a pooled dataset and having as a goal to be able to have country-based outputs, I would suggest the authors to explore the multilevel modelling techniques which are often used with the DHS data. Multilevel modelling techniques deal with the complex and clustered sampling data structure, and the hierarchical nature of the DHS data. Since multiple observations (women - level 1) are nested within clusters and multiple countries are nested within the same country, multilevel modelling deals with hierarchical structure of the data and the within and between cluster correlations of the observations, and therefore lead to more reliable results, while controlling for intra cluster correlation. The use of multilevel modelling though would need careful consideration, including a thorough check of the minimum number of units needed at each level and the construction of the group weights. Please explore previous studies and some books on the topic such as (just to give a few examples): Harvey Goldstein, Multilevel Statistical Modelling; Applied Multilevel Analysis: A Practical Guide. Jos WR Twisk. and the material from the Centre for Multilevel Modelling at the University of Bristol. Also, see a recent publication from the DHS Program on multilevel modeling using DHS surveys: a framework to approximate level-weights – DHS methodological reports 27.

**Response 5**: Thank you for your suggestion. Since we did not use a pooled dataset, multilevel modelling techniques was not required for our analysis. Each country's data was analyzed individually, and the results were displayed separately. Moreover, regarding the Ref (33) issue (new Ref 31), we have checked the URL again and it seems is it working fine. Please turn off any VPN or any other firewall that might block the Browser from opening such URLs. Thanks for your suggestion.

**Comment 6**: The language used is at bits too colloquial and sometimes there are mistakes (e.g. Line 152: The resident has kept a dichotomy: Urban and Rural.) Please revise the English, as the manuscript is not always up to the standards of an academic journal article.

**Response 6**: We appreciate your feedback on language usage. We have reviewed the entire manuscript again, edited it for language, and corrected any grammatical mistakes to meet academic standards. Thanks a lot for the feedback.

**Reviewer 2:**

**Comment 1**: It is well known that education plays a significant role in healthcare usage across countries, and education positively influences it. Many studies mention that like (Rizvi DS. Health education and global health: Practices, applications, and future research. J Educ Health Promote. 2022 Aug 25; 11:262. Doi: 10.4103/jehp.jehp_218_22. PMID: 36325224; PMCID: PMC9621358). What did this study add to what was already known on this topic?

**Response 1:** We appreciate your insightful comment. As we all know, education significantly influences healthcare usage, but there can be variations across different regions. Although this has been demonstrated in many studies, including "Health Education and Global Health: Practices, Applications, and Future Research," our study is unique in that it examines multiple regions simultaneously and identifies diverse patterns in each. No previous study has analyzed these countries together to draw such comprehensive conclusions. Additionally, we only included data from the DHS Program collected after 2017, with all our data from 2021 and onward, providing the most up-to-date information for this analysis.

**Comment 2**: The manuscript needs English editing.

**Response 2**: We appreciate your feedback on English usage. We have reviewed the entire manuscript again and edited it for language and clarity to meet academic standards. Thanks a lot for the feedback.

**Comment 3**: The title is informative but so long. It is better to be considered.

**Response 3**: We understand your concern about the title length. However, we believe it is essential to provide clear and comprehensive information to readers. The title aims to convey the study's scope and focus, ensuring that potential readers understand the study's relevance and context. We appreciate your understanding in maintaining the title's current form to achieve this clarity.

**Comment 4**: Line 51: it is better to write ‘’Education significantly influences healthcare utilization, positively impacting both primary and secondary education’’

**Response 4**: Thanks for addressing this. The line now changed in (line 51).

**Comment 5**: The aim is clear.

**Response 5**: Thanks for clarifying this.

**Comment 6**: What is the role of the researchers? Is that collected data from the Demographic and Health Survey (DHS)? You did not collect data by yourself?

**Response 6**: Thank you for highlighting the need for clarity regarding data collection. The data for this study was obtained from the Demographic and Health Surveys (DHS) Program, which collects data through standardized surveys in various countries. The researchers analyzed this secondary data, focusing on healthcare utilization among women. The DHS Program is authorized to distribute, at no cost, unrestricted survey data files for legitimate academic research (<https://dhsprogram.com/data/data-collection.cfm>). This information is now also added in (line 131) in the main manuscript.

**Comment 7**: Are you sure the variables are defined and measured appropriately? Why did you not use a flowchart to explain the variables?

**Response 7**: Thank you for this important point. We have rechecked everything and we ensure all variables are measured properly. For better understanding, an additional supplementary flowchart has been added in a diagram manner to make it comprehensive to understand the procedure we did. Thanks for your concern. This is cited in (line 201) and the supplementary file has been linked at (line 550).

**Comment 8**: The results need to be discussed from multiple angles and placed into context without being overinterpreted.

**Response 8:** We appreciate your suggestion and we precise the discussion section to demonstrate it from various perspectives and provide a broader context, ensuring a comprehensive understanding of the findings. A significant change has been made in the discussion section of the manuscript. Thanks for your consideration.
