# Supplementary material for: Does education sculpt healthcare choices? Exploring factors influencing healthcare utilization among female youths in eight low and lower-middle-income countries
Source: PLoS One. 2025 Mar 17;20(3):e0298782. doi: 10.1371/journal.pone.0298782 (PMC11913268; doi:10.1371/journal.pone.0298782)
Supplement: Attachment — Submitted filename: Response_to_reviewers_auresp_3.docx [file pone.0298782.s006.docx]

Academic editor comment

Comment 1: The discussion of results could benefit from a deeper analysis, particularly regarding the observed regional variations. For instance, the implications of this manuscript could be more explicit about the concrete policy consequences of the results. While the need to improve infrastructure and promote education is mentioned, more specific recommendations could be provided on how governments and organizations can address inequities in healthcare access. This could include proposals for specific programs tailored to each regional context or strategies to enhance the monitoring of certain public policies. It would be valuable to explore in greater detail the cultural, socioeconomic, or other factors that might have implications in these policies.

Response 1: Thanks. We have revised the manuscript and cited necessary references accordingly. Thank you for your suggestion.
